# Low HIV testing rates among tuberculosis patients in Kampala, Uganda

**DOI:** 10.1186/1471-2458-10-177

**Published:** 2010-03-31

**Authors:** Ibrahim Sendagire, Imke Schreuder, Mesach Mubiru, Maarten Schim van der Loeff, Frank Cobelens, Joseph Konde-Lule

**Affiliations:** 1 Kampala City Council, Public Health Department, P. O. Box 700, Kampala, Uganda; 2 Erasmus Medical Centre, Department of Virology, 3000 CA Rotterdam, The Netherlands; 3 Academic Medical Centre, Amsterdam, Center for Poverty-related Communicable Diseases, 1105 AZ, Amsterdam, The Netherlands; 4 Amsterdam Institute for Global Health and Development. P. O. Box 22700, Amsterdam, The Netherlands; 5 KNCV Tuberculosis Foundation, 2501 CC, The Hague, The Netherlands; 6 Makerere University School of Public Health, P. O. Box 7072, Kampala, Uganda

## Abstract

**Background:**

HIV testing among tuberculosis patients is critical in improving morbidity and mortality as those found to be HIV positive will be offered a continuum of care including ART if indicated. We conducted a cross-sectional study in three Kampala City primary care clinics: to assess the level of HIV test uptake among newly diagnosed pulmonary tuberculosis (PTB) patients; to assess patient and health worker factors associated with HIV test uptake; and to determine factors associated with HIV test uptake at the primary care clinics

**Methods:**

Adult patients who had been diagnosed with smear-positive PTB at a primary care clinic or at the referral hospital and who were being treated at any of the three clinics were interviewed. Associations between having taken the test as the main outcome and explanatory variables were assessed by multivariate logistic regression.

**Results:**

Between April and October 2007, 112 adults were included in the study. An HIV test had been offered to 74 (66%). Of the 112 patients, 61 (82%) had accepted the test; 45 (74%) had eventually been tested; and 32 (29%) had received their test results.

Patients who were <25 yeas old, female or unemployed, or had reported no previous HIV testing, were more likely to have been tested. The strongest predictor of having been tested was if patients had been diagnosed at the referral hospital compared to the city clinic (adjusted OR 24.2; 95% CI 6.7-87.7; p < 0.001). This primarily reflected an "opt-out" (uptake 94%) versus an "opt-in" (uptake 53%) testing policy.

**Conclusions:**

The overall HIV test uptake was surprisingly low at 40%. The HIV test uptake was significantly higher among TB patients who were identified at hospital, among females and in the unemployed.

## Background

Testing all patients routinely for HIV in settings with generalized HIV epidemics has the benefit of diagnosing the infection early and thereby preventing morbidity, mortality and sustained transmission through initiating prophylaxis and timely antiretroviral treatment (ART) [1-4]. Test uptake varies considerably from 12% to 98% across different settings and patients categories, but particularly with the testing approach used [5-8]. HIV test uptake levels of 12-62% have been reported when the test is offered using an "opt-in" approach [7,8], i.e. when patients are not tested unless they specifically request to. Uptake tends to be much higher (70-98%) when the HIV test is offered using an "opt-out" (or "provider-initiated") approach by which the HIV test is considered an integral part of the diagnostic procedures and patients are tested unless they specifically indicate that they do not want to [5,6].

HIV testing is particularly important in tuberculosis (TB) because mortality among HIV-infected TB patients is substantially increased unless co-trimoxazole preventive treatment or ART are provided [4,9-11]. TB was responsible for 1.3 million deaths among HIV negative incident cases in 2007 [12] and was the most common cause of mortality among HIV patients in sub-Saharan Africa [13,14], where HIV prevalence among tuberculosis patients ranged from 20 to 60% between 1995 and 2005 [15-18]. There is consensus that all TB patients should be tested for HIV infection in order to offer ART if positive and indicated [19]. So far few data have been published about HIV testing practices among TB patients in sub-Saharan Africa. Although some studies have shown high uptake levels [20,21], the WHO estimated only 0.5 million TB patients (37% of all notified cases) knew their HIV status in Africa in 2007 [12]. The reasons for these differences are largely unknown. HIV testing at the time of TB diagnosis is an excellent opportunity to make a new HIV diagnosis and get patients in care.

HIV testing in Uganda has evolved over the years. In the late 1990s and early 2000s only a small proportion of patients had access to HIV testing, mainly due to absence of the service and the policy of testing in place. Until recently, HIV testing in Uganda was based on a voluntary request from the patient to be tested ("opt-in" approach) at static testing centres and later on during outreach services [7,22]. In 2005 a new policy was adopted in Uganda that advocated for a shift to "opt-out" testing in order to scale up access to HIV testing and the blood samples are tested for HIV using a serial algorithm recommended by the Ministry of Health [23].

We studied HIV test uptake among TB patients in Uganda, one of the 22 countries with a high TB burden: Uganda notified 42,000 smear positive cases to the WHO in 2007 [12]. About 50% of TB patients, in Uganda, are estimated to be co-infected with HIV [24].

The main objectives of the study were to assess the level of HIV test uptake among newly diagnosed TB patients treated at the primary health care facilities in Kampala city, and the factors associated with this uptake. The secondary objective was to determine the factors associated with HIV test uptake at the primary care clinics.

## Methods

### Study setting

Kampala, the capital city of Uganda, has an estimated resident population of 1.4 million people [25]. The day population attracted by services and various economic activities nearly doubles the resident population. The city is divided into five administrative divisions [26].

Kiruddu, Kisenyi and Kiswa are three of the ten primary health centres operated by the Kampala City Council (KCC), offering mainly outpatient services including sputum smear microscopy and TB treatment in accordance with the DOTS strategy. These centres were purposefully chosen considering geographical representation. Kiruddu health centre is located in a semi-rural residential area. Kisenyi health centre is located in the middle of a densely populated low-income area. Kiswa health centre is located in an industrial area of the city. Each study site is located in a separate division of the city. These centres are level III health centres and are primary health care outlets. The health centres are faced with the challenges of heavy patient load, understaffing and periodic stock-outs of different commodities. Health services at these health centres are free of charge [27].

Complicated cases are usually referred to hospital. Some patients are diagnosed and initiated on TB treatment in hospitals, notably the main referral hospital, Mulago, and are referred to one of the KCC clinics to complete treatment. These patients are then registered for continuation of treatment at the KCC clinics. After registration at the clinic, follow-up and review of the patients is performed by KCC staff.

Kampala city alone, reports about one quarter of the over 40,000 TB cases notified annually in Uganda [28]. Between 39% and 50% of these cases are estimated to be co-infected with HIV [20,24]. The number of TB patients (all forms of TB) registered for treatment at the three KCC clinics was 580 in 2007, 68% (395/580) of which had smear-positive pulmonary TB.

### Study design

Between 1^st ^April and 17^th ^October 2007, we conducted a cross-sectional study about HIV testing practices among patients with pulmonary tuberculosis in three KCC clinics. All consenting patients aged ≥ 15 years with sputum smear-positive pulmonary TB, residing within Kampala city or 16 km from the city centre who were treated at the three clinics were interviewed using a structured questionnaire between 2 and 8 weeks following treatment initiation. Data were collected by trained interviewers using pretested questionnaires. The interviews were conducted during patients' treatment visits. The very sick patients; patients unable to consent due to mental illness; and those patients aged ≥ 15 to 18 years without a parent or guardian's consent were excluded from the study. Consecutive recruitment was done at each of the three study sites until a total pooled sample was realised.

Data were collected on whether an HIV test had been: offered, accepted, done and test results received. Data were also collected on various factors that might have affected uptake of an HIV test including age and sex; educational, marital and employment status; household wealth; type of health facility where the patient was first diagnosed and initiated on TB treatment; the type and attitude of the health worker offering the test; and patient knowledge about benefit of doing an HIV test and knowledge about preventing HIV infection. Smear positive TB was defined according to international guidelines [29].

A recent study had found a high HIV test uptake level of 98% among patients attending two large tertiary urban hospitals [5]. In another study among registered TB patients the HIV test uptake was 87% [21]. We reasoned that the HIV test uptake among TB patients attending urban health centres would be somewhere in the middle, at 92.5%. We put the level at 90%. A sample size of 96 patients was calculated to be sufficient to detect a test uptake of 90% with an error margin of 6% at the 95% confidence interval [5,21].

### Data handling and statistical analysis

Data were collected, entered into a custom programmed database using ASP.NET and stored in Microsoft SQL Server Express Edition database (Microsoft Software, Seattle, WA, USA). Statistical analyses were carried out using Stata statistical software, version 10 (Stata Corp, College Station, TX, USA).

The wealth of a patient's household was determined based on asset scores derived from the Uganda Demographic and Health Survey performed in 2000 [30]. A composite value of the household wealth was calculated as the total sum of the scores assigned to each asset owned or absent and divided into tertiles. Responses to question items about knowledge were each given a score leading to a dichotomous variable.

Excluded from the analyses were patients who were known to be HIV infected before the TB diagnosis. In the primary analysis, participants who had been tested were compared to those who had not. The secondary analysis compared the patients who had accepted an HIV test when offered to those who had not. Acceptance of the HIV test was calculated as the number of patients who accepted the test divided by the total number of participants offered the test. Overall uptake of routine HIV testing was calculated as the proportion of those who were actually tested relative to those who were eligible for the test. Associations between outcomes and explanatory variables were assessed by odds ratios in univariable analyses. Variables were entered in a multivariate logistic regression model if they showed a univariable association at p < 0.25, and included in the final model if they showed a significant association (at p < 0.05), or if they confounded the association with other variables in the model. Age and sex were included irrespective of their strength of association. All tests were done at the 5% significance level. In the secondary analysis, variables were examined for their association with having been offered an HIV test and with having accepted the test if diagnosed at the primary care KCC clinics.

### Ethical review

This study was reviewed and approved by the Makerere University School of Public Health (MUSPH), the Uganda National Council of Science and Technology (UNCST) and the Medical Ethics Committee of the Academic Medical Centre, Amsterdam. Informed consent was obtained from all participants. The consent forms were translated into Luganda, the commonly used local language. Patients between 15 and 18 years for whom it was not practically possible to get additional consent of the parents or guardians were excluded from the study.

## Results

A total of 120 patients on treatment consented to the interview. Eight were known to be HIV infected at the time of TB diagnosis, leaving 112 patients for the analysis (93%). Fifteen (13%) participants were recruited from Kiruddu, 61 (54%) from Kisenyi and 36 (32%) from Kiswa health centre. There were no significant differences between patients recruited from the various clinics in terms of sex (p = 0.56) and age (p = 0.54). Thirty-eight (34%) had been diagnosed with TB at the national referral hospital and then referred to the KCC clinics; the remaining 66% were both diagnosed with and started on TB treatment at the three clinics. The median age of the study patients was 30 years (range, 17-83). The majority was ≤ 30 years of age and 55% were male (Table 1).

**Table 1:** Socio-demographic characteristics of 112 smear-positive pulmonary tuberculosis patients included in a study on HIV test uptake, Kampala, 2007

Socio-demographic variable	N (%)
	
**Age**	
	
15 - 24 years	30 (26.8%)
25 - 34 years	46 (41.1%)
35 - 44 years	21 (18.8%)
45 - 54 years	10 (8.9%)
≥ 55 years	5 (4.5%)
	
**Sex**	
	
Male	61 (54.5%)
Female	51 (45.5%)
	
**Education level**	
	
None	7 (6.3%)
Primary (P1-P4)	15 (13.4%)
Primary (P5-P7)	38 (33.9%)
Secondary (S1-S4)	32 (28.6%)
Secondary (S5-S6)	10 (8.9%)
University	10 (8.9%)
	
**Marital status**	
	
Single	38 (33.9%)
Married	46 (41.1%)
Divorced/separated	21 (18.8%)
Widowed	6 (5.4%)
Co-habiting	1 (0.9%)
	
**Employment status**	
	
Unemployed	35 (31.2%)
Employed*	77 (68.8%)

*some form of employment including formal, informal and self-employed

**Figure 1:**
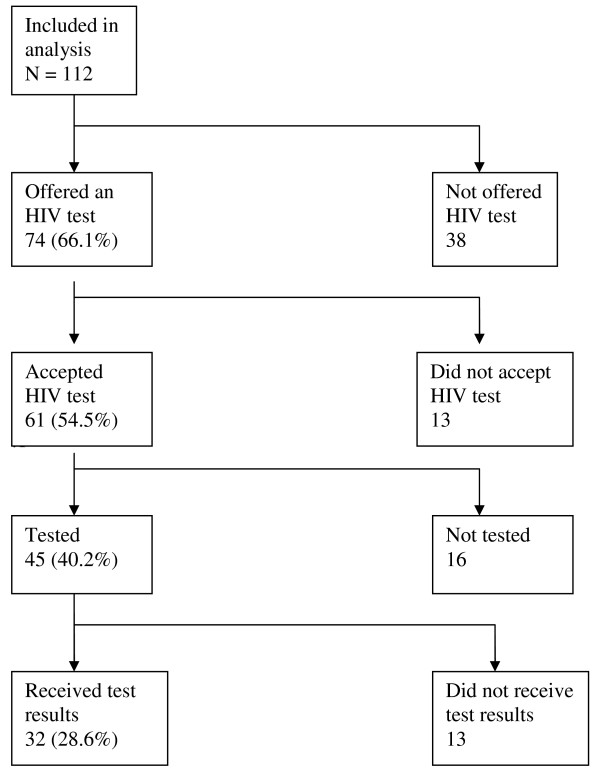
**Flow chart showing the participants included in the analysis**. Percentages refer to proportion out of 112.

Of 112 study participants, 74 (66%) had been offered an HIV test (Figure 1). Out of the 112 participants included in the study, 61 (55%) accepted to be tested. Overall 40% (45/112) of participants had been tested for HIV infection during or following the diagnostic process leading to their current treatment for TB. Thirty two (29%) received their test results.

When we stated our null hypothesis as "H_0 _= P is equal 90%" and the two-sided alternative hypothesis test as "H_1 _= P is not equal 90%", we estimated the HIV test uptake to be 40.2%; 95% CI 31.1%-49.3%. The test static, z, was equal to 10.8 and p was less than 0.001. We had sufficient evidence to reject the null hypothesis.

In the univariable analysis: the type of health facility where TB was diagnosed, a history of previous HIV testing and employment status were associated with having been tested (Table 2). Household wealth was not associated with being tested for HIV infection: middle tertile (OR 0.79 95% CI 0.31-2.04), upper tertile (OR 1.19 95% CI 0.47-2.97); data not shown. High education level was also not associated with being tested for HIV infection (OR 1.02 95% CI 0.47-2.17).

**Table 2:** Associations of testing uptake with baseline characteristics of 112 smear-positive pulmonary tuberculosis patients included in a study on HIV test uptake, Kampala, 2007

	Had HIV test		Univariable analysis		Multivariable analysis		
	Yes	No					
Variable	N (%)	N (%)	Unadjusted Odds Ratio	95% CI	Adjusted Odds ratio	95% CI	p-value
							
**Age**							
≤ 25 years	18 (48.7)	19 (51.3)	1	-	1	-	
26-33 years	12 (33.3)	24 (66.7)	0.53	0.20-1.36	0.10	0.02-0.45	
> 33 years	15 (38.5)	24 (61.5)	0.66	0.27-1.64	0.48	0.14-1.65	0.004
							
**Sex**							
							
Male	24 (39.3)	37 (60.7)	1	-	1	-	
Female	21 (41.2)	30 (58.8)	1.08	0.50-2.30	3.42	1.02-11.40	0.037
							
**Education level**							
							
Low	24 (40.0)	36 (60.0)	1				
High	21 (40.4)	31 (59.6)	1.02	0.47-2.17	-	-	-
							
**Employment status**							
							
Unemployed	9 (25.7)	26 (74.3)	1	-	1	-	
Employed	36 (46.8)	41 (53.2)	0.39	0.16-0.95	0.20	0.05-0.76	0.012
							
**Household wealth**							
							
Lower tertile	15 (40.5)	22 (59.5)	1				
Middle tetile	13 (35.1)	24 (64.9)	0.79	0.31-2.04	-	-	-
Higher tertile	17 (44.7)	21 (55.3)	1.19	0.47-2.97	-	-	-
							
**Health Facility at which TB was diagnosed**							
							
KCC clinic	16 (21.6)	58 (78.4)	1	-	1	-	
Hospital	29 (76.3)	9 (23.7)	11.68	4.61-29.62	24.22	6.69-87.73	<0.001
							
**Previous HIV testing**							
							
No	28 (56.0)	22 (44.0)	1	-	1	-	
Yes	17 (27.9)	44 (72.1)	0.30	0.14-0.67	0.15	0.05-0.48	<0.001

Adjusted odds ratios: adjusted for all other variables in the model. 95% CI: 95% confidence interval.

The likelihood of having been tested was not significantly associated with knowledge of two or more benefits of doing an HIV test (OR 1.44 95% CI 0.62-3.32), with knowledge of at least two ways in which HIV is transmitted (OR 0.73 95% CI 0.34-1.57), or with a perceived kind attitude of the health care worker (OR 1.55 95% CI 0.47-5.11).

In the multivariable analysis, the most important predictor of having been tested for HIV was whether patients had been diagnosed with TB at the referral hospital or at one of the KCC clinics (adjusted odds ratio (aOR) 24.22 95% CI 6.69-87.73, table 2). In addition, patients in some form of employment were less likely to have been tested compared to those with no employment (aOR 0.20 95% CI 0.05-0.76), as were patients with a history of previous HIV testing (OR 0.15 95% CI 0.05-0.48).

**Figure 2:**
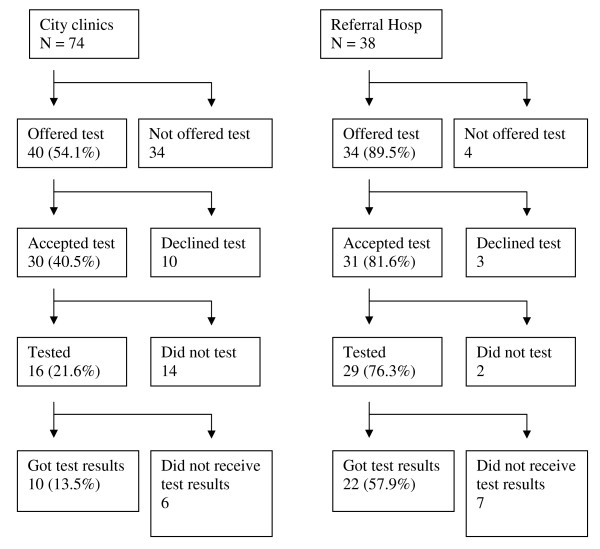
**Breakdown of patients according to health facility where tuberculosis was diagnosed**. Percentages refer to proportion out of 74 and 38 respectively.

Figure 2 shows test offer and testing uptake of patients according to the health facility where TB was diagnosed. Ninety percent (34/38) of patients diagnosed at the referral hospital had been offered an HIV test compared to 54% (40/74) of patients diagnosed at the KCC primary care clinics (p < 0.001). Acceptance of the HIV test was 82% (31/38) in the referral hospital and 41% (30/74) in the KCC clinics (p < 0.069). Of the 38 patients from the referral hospital who accepted to be tested, 76% (29/38) were eventually tested in the referral hospital compared to 16 out of 74 patients at the primary care KCC clinics (22%, p < 0.001).

Although more patients diagnosed at the referral hospital got their test results, 58% (22/38) compared to those diagnosed at the KCC clinics, 14% (10/74), this difference was not statistically significant (p < 0.343).

In the secondary analysis, variables were examined for their association with having been offered an HIV test and with having accepted the test if diagnosed at the primary care KCC clinics. Table 3 shows associations with having been tested once offered the test with predictor variables. Age, sex, employment status and history of previous HIV testing were not associated with HIV test uptake in the multivariable analysis.

**Table 3:** Associations of testing uptake with baseline characteristics of 30 smear-positive pulmonary tuberculosis patients diagnosed at Kampala City clinics included in a study on HIV test uptake, 2007

	Had HIV test		Univariable analysis		Multivariable analysis		
	Yes	No					
Variable	N (%)	N (%)	Unadjusted Odds Ratio	95% CI	Adjusted Odds ratio	95% CI	p-value
							
**Age**							
							
15 - 30 years	10 (58.2)	7 (41.2)	1	-	1	-	
≥ 31 years	6 (46.2)	7 (53.9)	0.60	0.13-2.67	0.42	0.08-2.31	0.308
							
**Sex**							
							
Male	8 (57.1)	6 (42.9)	1	-	1	-	
Female	8 (50.0)	8 (50.0)	0.75	0.17-3.26	2.39	0.39-17.9	0.384
							
**Employment status**							
Unemployed	13 (61.9)	8 (38.1)	1	-	1	-	
Employed	3 (33.3)	6 (66.7)	0.30	0.05-1.74	0.23	0.03-1.68	0.133
							
**Previous HIV testing**							
No	11 (64.7)	6 (35.3)	1	-	1	-	
Yes	5 (38.5)	8 (61.5)	0.34	0.07-1.65	0.21	0.03-1.41	0.088

## Discussion

In this study 40% of the tuberculosis patients who were treated at city clinics had been tested for HIV. Out of the 61 patients who accepted to be tested, 74% (45/61) were actually tested for HIV infection. This uptake level is low compared to recent studies that were carried out in Ugandan referral hospitals [5,6]. In line with this, we found a significantly higher HIV test uptake among patients who had been diagnosed with TB and started on treatment in the referral hospital and were subsequently referred to a city clinic than among patients who had been diagnosed with TB at the city clinic initially.

One possible cause for this difference is that patients received and admitted in the tertiary referral hospitals were more likely to have severe or long-standing illness, which may have prompted HIV testing as part of management of chronic illnesses. Perhaps more importantly, a policy of "opt-out" testing was already well established in the referral hospital at the time of this study. Although the "opt-out" testing policy had officially been introduced at national level, this had apparently not yet been fully implemented at the city council clinics. Not only had not all patients been offered an HIV test, only 75% had accepted it, which likely reflects an actual "opt-in" approach in these clinics.

These results indicate that in practice "opt-out" HIV testing was implemented at the referral hospital, but not yet at the KCC clinics. Moreover, 47% of patients diagnosed with TB in the city clinics had no HIV test done even though testing had been accepted. This suggests that problems in service provision played an additional role.

The low uptake at the city clinics is in line with a study conducted recently in rural Tanzania just before the roll-out of ART [8]. The uptake of 40% in our study was higher than the 12% in the Tanzanian study. The timings of these two studies were somewhat similar since in both settings provision of ART services was just beginning. This observation lends support to the view that, when the provision of ART services are absent or just beginning, the uptake of HIV testing is usually low.

The sex of patient, history of previous HIV testing and employment status, were other factors associated with doing an HIV test. Similar studies have shown similar associations with HIV test uptake [4,15]. A study carried out in an urban hospital found a high HIV test uptake among patients before admission [6]. Our study did not find a significant association between taking an HIV test and formal education nor income.

Unemployment was a factor found to be significantly associated with HIV test uptake in a study carried out in a public district hospital in south Ethiopia [31]. Our study findings are similar to these findings in this study possibly because both settings are from developing countries and both offering Directly Observed Therapy, Short Course (DOTS).

The study carried out in Tanzania found prior knowledge of VCT among others as important predictors of VCT completion [8]. Our study did not find an association between knowledge of benefits of taking an HIV test, transmission of HIV infection or cause of HIV infection and taking the HIV test.

Among the patients who were tested, 58% and 14% got their results from the referral hospital and the KCC primary care clinics respectively. The difference in getting test results was not statistically significant. The low level of getting test results in our study findings contrasts the results of a recent study that found that nearly all patients (95%) who had been offered HIV counselling and testing prior to admission had received their test results [6]. Our study findings show that a sizeable proportion of patients may not be entering into care because they don't know their HIV status. Improvement in the burden of TB or HIV may not be achieved if patients are not getting into care.

The true level of HIV test uptake among TB patients can as low as 31.1% and as high as 49.3%. Our study findings are similar to those of a recent study carried out in the referral hospital that found the test uptake to be low at 39% [20]. The implication here is that either the HIV test is not being offered to TB patients or the TB patients are not accepting to be tested after being offered the HIV test. There may be other operational factors yet to be determined that are responsible for TB patients not being tested.

Our study did not find any factor statistically significantly associated with HIV test uptake in the city clinics. However, patients with positive history of previous HIV testing were less likely to be tested again for HIV during their current treatment for TB, although this was not significant. This result may be due to the fact that the numbers were too small to give meaningful multivariable analysis.

Our study had some limitations. It was not designed to compare the two approaches of "opt-out" and "opt-in" HIV testing: we merely observed practices and found out that the patients started on TB treatment at the referral hospital had in fact been subjected to an "opt-out" system, whereas the city clinics appeared still to be following the "opt-in" approach. Other site differences e.g. availability of specialized care and co-localization of HIV services might indeed account for the improved offer and uptake of HIV testing at the referral hospital.

Study outcomes were obtained retrospectively and relied entirely on patient self-report. Patients seen at the referral hospital could have been sicker, had a longer day at the hospital or HIV testing policy was implemented better which may all result in better recall about HIV testing offer and uptake. Although the total pooled sample was representative of the city population as practically as was possible, the fact that study sites were purposefully chosen and not randomly selected could have introduced some form of selection bias in our findings. The sampling strategy may have resulted in a possible selection bias because random sampling of the sites was not done.

The sample size of 112 patients had high power to detect our primary outcome of the HIV test uptake among patients receiving care in urban health centres and the factors associated with this uptake. The sample size was however, not powered enough to pick the secondary outcome of the factors associated with the HIV test uptake among the patients diagnosed and registered for treatment at the KCC clinics when taken alone. Nonetheless this study illustrates the complexity of not only offering HIV testing in TB clinics, but also the challenge of ensuring that all steps from acceptance to testing to receipt of the results in a primary health care setting are followed. The study further demonstrates that operational research in HIV testing at the primary care level as recommended by the WHO is feasible in resource limited settings.

## Conclusions

The overall HIV test uptake was surprisingly low at 40%. The HIV test uptake was significantly higher among TB patients who were identified at hospital, among females and in the unemployed. We recommend that further studies be done in which the two approaches are compared in similar settings. We also recommend further operational studies into the barriers to implementation of HIV testing in the primary health care facilities.

## Competing interests

IS and MM are employees of Kampala City Council.

None of the other authors have any competing interest to disclose.

## Authors' contributions

IS: participated in the conception and design of the study; writing of the study protocol; acquisition of the data, supervision of the study; analysis; interpretation of data; drafted the manuscript. IS: participated in the conception and design of the study; writing of the study protocol; acquisition of the data, analysis and interpretation of data; drafting the manuscript. MM: participated in the conception, design of the study, study supervision and reviewing of the protocol; reviewed the manuscript critically for intellectual content. MS: participated in the conception and design of the study; reviewing of the protocol; analysis and interpretation of data; reviewed the manuscript critically for intellectual content. FC: participated in the conception and design of the study; reviewing of the protocol; analysis and interpretation of data; reviewed the manuscript critically for intellectual content. JK: participated in the conception and design of the study; reviewing of the protocol; analysis and interpretation of data; reviewed the manuscript critically for intellectual content.

All authors gave final approval of the version to be published.

## Pre-publication history

The pre-publication history for this paper can be accessed here:

http://www.biomedcentral.com/1471-2458/10/177/prepub
